# Engagement, Passion and Meaning of Work as Modulating Variables in Nursing: A Theoretical Analysis

**DOI:** 10.3390/ijerph16010108

**Published:** 2019-01-03

**Authors:** Juan Gómez-Salgado, Yolanda Navarro-Abal, María José López-López, Macarena Romero-Martín, José Antonio Climent-Rodríguez

**Affiliations:** 1 Nursing Department, University of Huelva, 21007 Huelva, Spain; jgsalgad@gmail.com; 2 Safety and Health Posgrade Program, Universidad Espíritu Santo, Guayaquil 091650, Ecuador; 3 Department of Social, Developmental and Education Psychology, Faculty of Education Science, University of Huelva, 21071 Huelva, Spain; 4 Department of Clinical and Experimental Psychology, Faculty of Education Science, University of Huelva, 21071 Huelva, Spain; mjlopez@uhu.es; 5 Red Cross Nursing University Center, University of Seville, 41009 Sevilla, Spain; mromero@cruzroja.es; 6 Faculty of Health Sciences, Isabel I University, 09003 Burgos, Spain; jose.climent@dpsi.uhu.es

**Keywords:** nursing, positive psychology, engagement, passion, meaning of work, care

## Abstract

Nurses are continuously exposed to stressors and psychological and physical risks that can negatively influence their daily work. These adverse psychosocial circumstances, accompanied by the poor self-perception of health, well-being, and quality of life, may trigger burnout. The positive psychology approach pursues a growth of passion at work, increased job satisfaction, and occupational health, both mental and physical, for the optimal performance of the nursing role. In this way, a theoretical analysis was conducted to describe the basic constructs of positive psychology, elements such as engagement, passion at work, centrality, and meaning of work, which could act as protective factors in the nursing profession. The results show that if health professionals are not physically involved, cognitively alert, and emotionally connected, they may not offer the quality care patients require. Positive psychology helps nurses in facing their complex reality and relevant daily activities in order to provide quality care. These efforts towards a humanist nursing care in which professionals are able to care for the others as well as themselves should be made.

## 1. Introduction

Health care professionals constitute one of the professional groups most frequently exposed to psychosocial risks at work. More specifically, nursing professionals often compromise their health by frequent, ongoing, and persistent exposure to physical, chemical, biological, mechanical, ergonomic, and psychosocial risks of different scope and nature [1]. Furthermore, they are subject to idiosyncratic working conditions, such as intensive and rotating work shifts, which play a role in the development of psychological diseases [2].

Health care professionals frequently experience symptoms that are related to working conditions that influence their psychological well-being such as high levels of stress, anxiety, emotional overload, or compassion fatigue. These arise, mainly, as a result of the nature of their work and the characteristics of their practice environment. Also, patients’ complexity triggers the particular needs of health professionals who provide care. Efforts should be made to address these needs and to ensure that health professionals feel prepared, equipped, and supported to care for complex patients [3]. These adverse psychosocial circumstances are also related to lower levels of self-perceived health and well-being and to general quality of life [4].

As Ceballos, Valenzuela, and Paravic pointed out, it is important that health institutions and governments consider psychosocial risk factors, enabling the development of prevention plans and providing incentives to nursing professionals by previously identifying those who are particularly subject to these risks [2]. As a result of these considerations, in recent years, concern about health professionals’ occupational health has prompted the development of studies on pathologies and personality traits that are linked to the professional practice. A range of recent studies analyzed the burnout syndrome [5] and its relationship with some personality traits, such as resilience [6] or capacity for empathy [7].

The data obtained in recent research highlight nurses as a professional group that is particularly exposed to psychosocial risks, something that draws attention to the need to address this fact as an issue that causes suffering and personal and professional hardship. They also consider how this causes significant economic losses for health care institutions, which in turn have a direct impact on the quality of care that is provided to patients. 

The origin of positive psychology dates back to the 20th century, when it was first defined as the scientific study of positive subjective experiences, positive individual traits, institutions supporting its development, and intervention programs promoting people’s quality of life by helping to reduce the incidence of mental health disorders [8]. However, as Salanova, Llorens, and Martínez pointed out, the concept had already been used by Maslow in 1954 in his description of motivation and personality, providing a more conceptual approach within the humanistic tradition, without taking into account a research basis [9].

In short, positive psychology is based on applying the foundations of psychology to different fields of action. Its fundamental basis, as its name suggests, seeks ‘the positive aspects’ in events and people instead of placing the emphasis on those areas presenting deficits or disorders. This perspective constitutes a tradition in 20th century psychology.

According to Lomas and Ivtzan, the development of this approach presents two distinct moments. There was a first stage in which positive psychology emerged as a movement opposed to traditional psychology, which seemed to focus on the more negative aspects of human beings, such as disorders, traumas, and problems. At this stage, positive psychology described a more simplistic concept based on a hedonist approach in which the emphasis was only on positive aspects, such as positive emotions, character strengths, or flow [10]. This was followed by a second wave in which authors such as Wong talked about ‘positive psychology 2.0’. In this case, both positive and negative aspects are part of the same dimension, where both elements are connected through a process of thesis-antithesis-synthesis [11].

Positive psychology is useful in preventing nurses’ burnout. This approach proposes improving nurses’ emotional competences, such as taking an optimistic view or acquiring stable personality skills instead of just decreasing the environmental factors of burnout [12]. Personal resources that comprise positive psychology, such us optimism, stable personality, and emotional competence, are related to the interaction between nurses and their working environment. They have been identified as factors of appraisal, adaptive strategies, and subjective well-being [13].

The purpose of this work was to describe the basic constructs of positive psychology that are related to the nursing profession. We aimed to identify the consequences it can directly have on the professionals’ quality of life, on the quality of the delivered nursing care, and on patient safety. The literature describes some risk and protective factors that are influential in professional performance, as well as in the subjective well-being and quality of life of professionals. However, these factors are directly related to organizational variables, which are, in many cases, beyond the control of professionals, and are more directly related to extrinsic motivators. In recent years, the scientific community has shown interest in the analysis of variables that are more related to the values developed throughout the life history of people, such as passion, centrality, meaning through work, or others, such as engagement, which are oriented towards the positive characteristics of professional performance.

## 2. Materials and Methods

**Figure 1:**
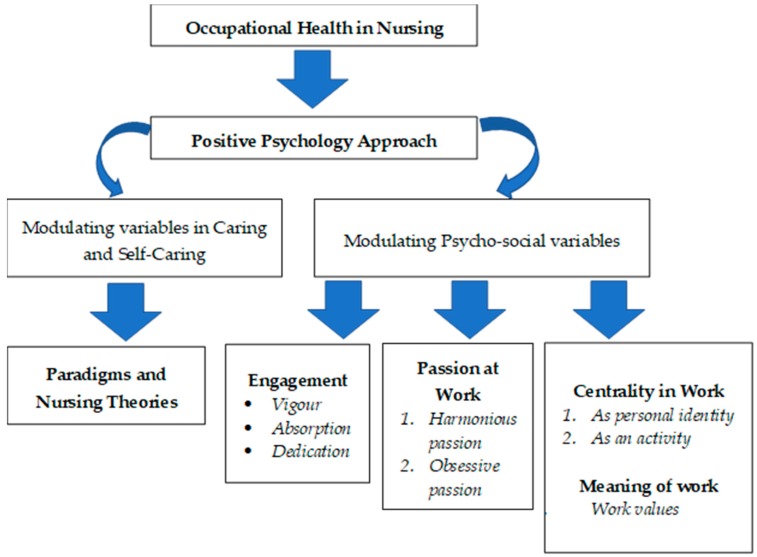
Concepts flowchart.

A theoretical analysis was conducted. First, core nursing concepts (caring and self-caring) were described, as they are determinant for nurses’ work overload and health. Subsequently, the positive psychology variables were analyzed to clarify their effect on nursing professionals and on the quality of care. The concepts flowchart is represented in Figure 1. Afterwards, the variables related to positive psychology were described, especially those that allow the prevention and mitigation of the health consequences that they may have for the nursing profession. Its study and analysis allow for generating organizational strategies that directly and indirectly impact in both the professional projection towards the patient (care) and the own projection towards the self (self-care).

## 3. Results

### 3.1. Care and Self-Care in Nursing

#### 3.1.1. The Inner Good of Nursing

Caring has evolved and changed throughout history in many aspects, while maintaining its essence. Since prehistory, the role of the caregiver has been assigned to women, who in most cases relied on their own intuition. Over time, civilizations have become aware of the importance of care in determining health. Caring evolution has been conditioned by different factors, such as religion, military conflicts, relationship with medicine, or the role of women in society, until finally obtaining professional recognition [14].

Caring is considered as the ‘inner good’ of the profession [15], and nurses, as part of the nursing metaparadigm, must ensure its presence in all caring areas [16]. Caring has been extensively studied and deeply discussed by theoretical nurses, each of whom brings a nuance from his/her own perspective.

Kérouac and Ducharme described the principles that guide nursing professionals’ everyday activities, supported by and subject to the code of ethics [17]:

- Nursing professionals must assist not only the individuals as such, but also their families, groups, or communities.
- Patients’ opinions must be respected at all times, accepting their values and beliefs, dignifying them, and ensuring a comprehensive view of the person.
- The art of caring is a construct that goes beyond the simple automation of the nursing practice; it implies professional performance from a comprehensive perspective by applying specific knowledge and principles.
- In addition to the specific contents of the nursing field, caring must integrate knowledge from other fields, such as mathematics, statistics, psychology, and sociology, among others.
- Disease prevention and health promotion are highlighted objectives of nursing, acting as helpers for the restoration of health and reducing, to the extent possible, the complications that may arise.
- Each health professional has a specific role within the multidisciplinary team, and individually contributes to the patient’s health-disease process.
- Patients have an active role, participating in their own self-care.

#### 3.1.2. Nursing Paradigms. Conceptions of Care

There are plenty of theories and models attempting to interpret the nursing metaparadigm. Efforts have been made to establish connections between these theories that are based on the beliefs, values, principles, laws, and methodologies they have in common in order to classify them. Raile and Marrineer established a categorization according to the models’ level of development (philosophies, conceptual models, theories, and middle-range theories) [18]. Similarly, Colley established a classification of theories according to their function (descriptive, explanatory, predictive, and prescriptive) [19]. The most common and most widespread classification follows a distribution by paradigms, defined as the line of thought or way of seeing the world that has an impact on all fields at a certain moment [20].

Years later, Kérouac and Ducharme described three opposed paradigms that influenced different conceptions of the art of caring and which have served to organize the nursing theoretical knowledge [17]. These paradigms are compared in Table 1.

### 3.2. Engagement

Within this framework, engagement is attracting interest and gaining relevance in scientific publications. Engagement was first mentioned by Khan, who analyzed the attitudes of employees in organizations that dealt with vulnerable population (the sick, the elderly, or low-income groups) [21]. Under equal conditions, and excluding the variables that could mediate these attitudes, some employees demonstrated a highly energetic behavior and a high degree of motivation towards their professional performance. As a result, problems within the organization and the most frequent coping strategies to solve them were described [22]. In these terms, the concept of engagement was defined as the behavioral attitude of using all of the individual’s components and being expressed through all of them, i.e., physical, cognitive, emotional, and psychologically, during the performance of the professional role [23]. Therefore, people express some level of engagement when they are physically involved, watchful to cognitive level, and in emotional connection.

Kahn identified three psychological conditions that modulate the presence of engagement: (a) psychological meaningfulness: sense of job performance satisfaction; (b) psychological safety: confidence in one’s job involvement without fear of negative consequences; and, (c) psychological availability: people’s physical resources to engage the self in work performance [21]. Salanova and Shaufeli identified three psychological conditions manifested by people with engagement: (a) satisfaction: the work itself is considered significant, constituting a challenge for the person; (b) safety: the workplace is considered reliable, safe and predictable, generating a positive working environment for the person; and, (c) availability: the required physical and psychological resources to develop the profession are at hand and available to improve the occupational role performance [24].

Engagement integrates three components [24]:

- Vigor (behavioral-energy component): is considered the variable opposed to the emotional exhaustion that is caused by the burnout syndrome. It is characterized by high levels of energy and mental resilience, persistence, right intention, and attitude towards investing efforts, even when facing difficulties.
- Absorption (cognitive component): is considered the variable opposed to the personal frustration caused by the burnout syndrome. It is characterized by facing difficulties to disconnect from work and by being so fully concentrated that time seems to pass quickly.
- Dedication (emotional component): is considered the variable opposed to the cynicism caused by the burnout syndrome. It is characterized by work involvement, enthusiasm, and satisfaction, as well as motivation towards achievement.

Within the nursing field, work engagement requires trust and professional autonomy. When high levels of work engagement are reached, contagious personal initiative occurs, hospital mortality decreases, and organizations’ financial profitability significantly increases [25]. Work engagement affects nurse performance resulting in patient-centered care, efficacy, and quality of care [26]. 

Engagement is adaptable, as it is subject to modifying factors. A study that was conducted by Dasgupta about factors affecting work engagement in nursing found out that perceived organizational support, leader–member exchange, team-member exchange, and workplace friendship all relate positively to work engagement. The nursing role stress negatively relates to work engagement [27]. Keyko, Cummings, Yonge, and Wong identified 77 influencing factors of work engagement. They were categorized into six themes: organizational climate, job resources, professional resources, personal resources, job demands, and demographics variables [28].

Rewards, organizational and supervisory support, and job characteristics contribute to establishing work engagement among nurses [29]. A positive work climate, social support from the organization, and the influence of supervisors through leadership styles are factors that stand out as fostering engagement [26].

### 3.3. Passion at Work

As Vallerand et al. [30] pointed out, passion is a topic of interest in the field of philosophy, and many definitions and concept analyses have been provided by experts, which can be grouped into two different approaches. On one hand, the emphasis on the idea that passion involves the loss of reason and control. Authors such as Spinoza (1632–1677) proposed that acceptable and healthy thoughts are originated by reason, while inappropriate thoughts are derived from passion. In this way, passion controls peoples’ lives and makes human beings slave of their own suffering. On the other hand, a more positive perspective of passion considered it as a beneficial variable when emotions are under control. Authors such as Descartes (1596–1650) in ‘The Passions of the Soul’ (1649–1972) defined passions as strong emotions with behavioral tendencies that can be positive [30].

In the field of psychology, the concept of passion is frequently linked to motivation, and it is related to certain concepts, such as creativity, love, etc. Vallerand et al. [30] defined passion as a strong tendency towards an activity that people like, that is important for them, and in which they invest time and energy. They proposed a dualistic approach in which there are two types of passion: obsessive passion and harmonious passion. Both impact differently on the individuals’ identity, which is understood as their personality characteristics, their experiences, and the relationship between these two concepts.

The features of both concepts regarding the control of the activity, its affective consequences, and the consequences for job performance are evident:

(1) Harmonious passion is the result of the internalization of the autonomous activity into the individual’s identity. The accomplishment of this task is freely accepted and it is not mediated by contingencies other than the person’s own motivation. The participation is voluntary, developing a sense of will and personal support. Therefore, the activity holds a significant place in the life of the person, but it allows for harmonizing activities with the other life areas. Harmonious passion will lead to more positive and less negative affect during the time dedicated to the task.
(2) Conversely, obsessive passion is the result of an internal level of control of the activity developed. The person feels self-imposed pressures as well as those from others. A sense of pathological dependence is developed, which consequently brings the impossibility of avoiding the performance of the desired task. Therefore, the activity that was initially a pleasure becomes an obligation that must be under control.

Individuals suffer intrapersonal and/or interpersonal pressures either because of certain contingencies or because their own emotions generate a feeling of dependency that leads to an uncontrollable need to participate in the activity. Thus, although they are excited about participating in the activity, they feel immersed in a sense of obligation and, then, the activity becomes the controlling agent. These individuals focus their lives on the activity, which holds a prominent place in their identity and brings along a lack of compatibility with other aspects of their lives. This circumstance not only creates an intrapsychic conflict, but also many interpersonal ones, making it difficult to reconcile one’s everyday labor duties with other life areas. Subsequently, these people suffer an internal compulsion that leads them to participate in the activity even when they should not, and this may cause a conflict between the passionate activity and their participation in other tasks [31]. Thus, a cognitive dissonance is usually found between what they should be doing and what they actually do, thus justifying the time devoted to these activities. They feel emotionally trapped by the activity, even knowing its consequences are not beneficial and that these can even bring negative consequences. They consider that they should perform the activity and, eventually, the activity is in control of their lives.

Nursing is understood as a vocational profession. Vocational workers identify themselves with certain meaning and ideology that, in the case of the nursing professional, is caring [32]. Thus, due to vocational caring, nurses are predisposed to develop a high passion for work. When working with passion, professionals are more effective and health teams are more stable, thus reducing staff turnover [33].

Passion at work can be promoted. Li, Zhang and Yang found a positive association between leaders’ and workers’ passion at work: leader’s work passion was transferred to employees via emotional contagion. Leaders may act as role models in fostering passion at work [34]. Luo, Bai, Min, Tamg, and Fang proposed training opportunities and personal development opportunities in order to increase passion, enthusiasm, and satisfaction with work [33]. According to Bushardt, Beal, Young, and Khosla, critical thinking and mindfulness training could be effective strategies for preventing the negative effect of passion excess [35].

### 3.4. Centrality and Meaning of Work

Work centrality has been defined from different perspectives. It can modulate job matching and, therefore, lead to greater happiness in the workplace. The Meaning of Work (MOW) research team has studied this concept in depth since 1987. They describe it as the perception of each individual regarding their work, and the value, relevance, and presence that work represents in their lives [36]. These authors make this construct operational and distinguish two types of centrality: absolute and relative. Absolute centrality relates to the general significance of work for each person. Relative centrality relates to the importance that each individual attributes to work, as compared with other life aspects, such as family, leisure, religion, or community and social relationships [36]. Kanugo identified two main elements in work centrality: work-role centrality, considered as the socially accepted belief about the meaning of work; and, work centrality, as an occupation related to other human areas [37].

Values are also a core concept in the meaning that people attribute to work. Values that are associated to work, in addition to normative beliefs and the central role that work plays in one’s life, are essential for a whole understanding of the concept of meaning of work. Work values refer to expectations and rewards that were obtained through working. Hoppok and Super studied the relationship between work satisfaction and certain aspects, such as salary, schedules, promotion, or diversity [38]. Super described work values, as the set of objectives that people expect to achieve from working and that motivate them to work, could be intrinsic or extrinsic [39]. According to Zytowski, work values could be considered as mediating concepts between affection and the external objects that offer a similar satisfaction [40]. Pryor defined work values as qualities or rewards that are expected to be obtained from working (money, safety conditions, altruism…) [41].

Nurses deal with emotionally stressful situations involving suffering, pain, sense of loss, and other hard feelings on the daily basis. To cope with these vulnerable situations, nurses expect a reward that goes beyond money or job stability. Finding meaning in work is also essential for coping with stressful situations inherent to the nursing work [42]. The meaning of work has been explored in nursing. Lee concept analysis revealed four main attributes: experienced positive emotion at work, meaning from work itself, meaningful purpose and work goals, and work as a part of life that contributes towards a meaningful existence [43].

Nurses have described the key elements that bring meaning into their work: relationships with each other and with the patients, a commitment to compassionate caring, personal identity with the profession, and mentorship that they received as students [42]. Pavlish and Hunt exploratory study revealed that connections, contributions, and recognition were the requirements for a meaningful work for nurses. Participants described learning-focused environment, teamwork, constructive management, and time with patients as facilitators of meaningfulness and task-focused environment, while considering stressful relationships and divisive management as barriers [44]. Spending time with the patient also improves the meaning of work, as well as the relational aspects of caring [45].

## 4. Discussion

The theoretical analysis revealed engagement, passion at work, centrality, and meaning of work as positive psychology variables that affect nursing professionals and quality of care.

Some studies argued that engagement is a consequence of certain situations, while others claim that it is engagement that influences these situations [24]. The variables, both personal and situational, which can help to develop engagement, have been analyzed. Regarding the situational variables, personal and job resources, or even emotional contagion, can be considered as triggering causes of engagement. Other situational variables, however, can be influenced by engagement: attitudes towards the specific task or institution, actual performance of such tasks, and self-efficacy. Regarding the personal variables, Hakanen, Bakker, and Schafeuli highlighted proactivity, motivation towards achievement, responsibility and decision-making ability, problem solving, and ability to address risks [46]. Salanova and Shaufeli considered that one of the basic characteristics of engagement is assertiveness towards complex situations, as it favors the perception of self-efficacy regarding job performance [24].

More specifically, in the nursing field, some psychological characteristics have been identified as relevant to face job demands: emotional intelligence, level of resistance to situations, and coping strategies. A research conducted by Bakker and Sanz-Vergel with primary care nurses showed that the sense of self-efficacy and optimism was highly related to engagement in situations where high emotional overload and low-time pressure were perceived [47]. Wan, Zhou, Li, Shang, and Yu aimed at identifying work engagement predictors in registered nurses. Findings showed that nurses’ age, job characteristics, and practice environment were significantly related to work engagement [48]. Engaged employees have also proven to be healthier and more efficient. Therefore, effective management of excessive workload, higher levels of autonomy, and greater job support are required to minimize the impact of job stressors [49].

Regarding passion for work, Cazares and García carried out research aiming to analyze the relationship between passion for work, resilience, and quality of professional life in hospital nurses. The results showed a professional life quality ranging from low to high. It concluded that nurses are satisfied with their job and with providing care [50]. Another study that was carried out by Trépanier, Ferne, Austin, Forest, and Vallerand analyzed the role of passion at work in health deterioration and in motivation towards work, according to the job demands-resources model. When considering the dualistic approach to passion, the objective of this paper was to analyze the correlation, on the one hand, between harmonious and obsessive passion and job demands and burnout/commitment and, on the other hand, the correlation between harmonious and obsessive passion and job resources and burnout/commitment. The results showed that both types of passion partially modulate the relationship between job demands and commitment, while harmonious passion influences the relationship between job resources and burnout/commitment [51].

Although involvement, engagement, and work centrality have been used as the same concept in the literature, there are conceptual differences between them [52]. Salanova stated that centrality is the place that work occupies in one’s life, as perceived by the person. Involvement and commitment to work relate to cognitive, affective, and emotional responses that are associated to the labor activity, and also their attitudinal and behavioral implications [53]. An analysis that was made by the MOW group on work values identified two integrating concepts. On the one hand, valued work outcomes represent the motivation for working, the goals that each individual aims to achieve, this is, the main reasons why people go to work. On the other hand, the importance of work goals is the relevance of work perceived by individuals and it should be understood as the updating or correction of values in a particular job.

Involvement, engagement, and work centrality have been defined as different concepts in literature. García, Pinazo, and Carrero conducted a theoretical analysis of the empirical research involving these concepts. They concluded that, although they have been used and operationalized separately, they share a common theoretical basis, which is the importance of work [52]. Salanova, Gracia, and Peiró description of job involvement and commitment to work includes emotions and cognitive responses, as it focuses on attitudes and behaviors that are derived from the job performance. According to these authors, centrality refers to the relevant position that work occupies in someone’s life, without the emotional connotations [53].

Regarding work values, the MOW group distinguished between the valued results of the work and the importance of work-related issues [36]. Work values are considered to be powerful motivating elements [54]. The valued results of the work are the reason for working. They guide people in their job sphere as they are the outcomes expected by workers. The importance of work goals depends on the workers’ perception about their job. They are concrete values and outcomes that workers obtain from work.

A traditional classification grouped these aspects into social, extrinsic, and intrinsic to the work itself. Salanova, Gracia, and Peiró highlighted that intrinsic values focus on the individual’s actual working activity (aspects related to the content of the work), from which the person exercises internal control [53]. This activity is a purpose in itself, and is an expressive, valuable, and satisfying activity for the person. On the contrary, extrinsic values do not focus on the work performed, but on the context of work, which exercises external control over the person. This work, which was performed to obtain some benefit, is not a purpose in itself, but a means for achieving other goals. Intrinsic aspects refer to characteristics of the work activity, i.e., whether it offers varied tasks, responsibilities, opportunities to develop competences and skills, or provides opportunities to learn. Extrinsic aspects refer to working conditions, such as schedule, job security, and earnings. Finally, it is worth distinguishing the social aspects of work. Thus, the acquired social status and relationships with colleagues are also valued aspects by workers.

## 5. Conclusions

Health professionals, especially nurses, experience high levels of stress, anxiety, emotional overload, and/or compassion fatigue, mainly as a result of the nature of their work and workplace. Mental health, job satisfaction, and working conditions can influence, either as protective or as damaging factors, their professional performance. As it is a collective that deals with people’s health, any professional error that is caused by psychosocial distortion could result in damage to patients and even death or disability.

Positive psychology is regarded by many authors as the cornerstone for improving the quality of employees’ lives, and it can also be a key tool for improving nursing quality of care and patient safety. For people to be fully developed in their professional performance, favorable satisfaction with their bio-psychosocial dimensions is necessary, and it is here that this branch of psychology is particularly relevant.

Strategies to promote engagement, increase workers’ satisfaction with their activity, improve safety conditions, and generate a positive work environment and sufficient availability of physical and psychosocial resources for the performance of the role should be implemented in order to prevent burnout.

Positive psychology helps nurses to face their complex reality and relevant daily activities, also contributing to providing quality care. We must direct our efforts towards a humanist nursing care, in which professionals are able to care for the others as well as for themselves. If the professionals are not physically involved, cognitively alert, and emotionally connected, they may not offer the quality care that patients require.

### Limitations and Future Research Work

People management policies implemented in organizations tend to centralize their importance in a culture of prevention regarding certain risk factors. Although, in recent decades, emphasis has been put on the importance of psychosocial factors, these strategies are based on the targeting of interventions in situations of stress and their consequences, such as the burnout syndrome or anxious-depressive disorders. The main limitation that was found in this study was the shortage of papers where intervention strategies were orientated to the development of values that promote attitudes included in the field of positive psychology. There are studies that propose a descriptive analysis diagnosis of the organizations where nurses perform their profession, and studies that offer programs aimed at interventions in pathological situations. A line of future work could be the implementation of intervention programs in healthcare organizations where values, such as engagement, passion, centrality, and meaning of work, are developed, conducting an analysis of the benefits of these programs.

## Figures and Tables

**Table 1:** Paradigms influencing nursing theories.

Paradigm	Ideology	Nursing Metaparadigm	Representative Authors
Categorization 1900–1950	Proposes dividing phenomena into categories or groups exploring the cause of the phenomena. It suggests a bio-medical approach, and a disease/public health orientation. The nurse has the role of planning, organizing, evaluating, and coordinating caring (understood as disease mitigation). Influenced by positivism.	Environment: it is independent of the person and something that can be controlled and manipulated. Health: absence of disease. Individual: a whole conformed by the sum of their dimensions, with a passive role in care. Care: focused on controlling the disease.	Florence Nightingale, Environmental Theory. Mother of modern nursing: she believed that through the manipulation of the environmental elements, nurses could facilitate patients being healed by nature. The patient is considered as a passive being in the reception of care.
Integration 1950–1975	Presents a person’s orientation, acting collaboratively, refusing the passive conception of the patient, and understanding the individual as a bio-psycho-socio-cultural-spiritual being. The nurse is an advisor, a co-worker. Influenced by psychology.	Environment: context in which the person lives with positive and negative stimuli and adaptation reactions. Health: ideal to achieve. Individual: bio-psycho-socio-cultural and spiritual being, greater than the sum of its dimensions. Care: efforts to maintain the person’s health in all its dimensions.	Virginia Henderson, Model of Independence. The individual was considered as an integral being with 14 basic needs that should be met as independently as possible to achieve their independence. Help was only needed in case of lacking strength, knowledge, or willingness. The role of the nurse depends on the level of dependence: substitute for the patient (when the nurse performs the action), assistant of the patient (when the nurse acts with the patient), and companion of the patient (when both collaborate, but the professional monitors). Dorothea Orem, Self-Care Deficit Theory. This theory is focused on the pursuit and achievement of the highest level of patient self-management. The main concept of the theory is self-care, that it is understood as the set of unintended actions a person performs or would perform to control the internal and external factors that can jeopardize their lives and further development. When self-care demand exceeds the capacity of the care agency, the figure of the agent of dependent care and the nursing systems would come into action (fully compensatory, partially compensatory, and education-support systems).
Transformation 1975–present	Supposes openness towards the world and the universe where everything is connected. Nurses accompany patients by sharing their experiences, collaborating with the individual in their care as a unique, personal, and individual type of care. Influenced by the general systems theory.	Environment: universe to which the person belongs. Health: a value and an experience according to the perspective of each person. It goes beyond the disease. Individual: an indivisible whole in continuous and mutual relationship with the universe. Care: focused on well-being. Cultural values, beliefs, and convictions of the person are recognized.	Jean Watson, Theory of Transpersonal Caring. She highlights the importance of the nurse-patient relationship based on the principles of empathy and warmth. Madeleine Leininger, Theory of the Cultural Care Diversity and Universality. She emphasized the importance of culture in care based on the holistic conception of the person.
